# P16 Using virtual reality to help manage needle phobia in juvenile idiopathic arthritis

**DOI:** 10.1093/rap/rkab068.015

**Published:** 2021-10-19

**Authors:** Sarah McCarthy, Stephanie Jones, Alexandra Tabor, Jon Packham

**Affiliations:** 1 Keele University Medical School, Keele, Staffordshire, United Kingdom; 2 Midlands Partnership NHS Foundation Trust, Stoke on Trent, United Kingdom; 3 University Hospital of the North Midlands, Stoke on Trent, United Kingdom; 4 Academic Unit of Population and Lifespan Sciences, University of Nottingham, Nottingham, United Kingdom

## Abstract

**Case report - Introduction:**

Needle phobia is a common issue amongst children, particularly with long-term illnesses requiring repeated interventions. Psychological interventions such as coping strategies and distraction techniques can be effective in many of these children, but is not universally successful. Recent research has reportedthat the use of virtual reality (VR) headsets as a distraction technique can be effective at alleviating both pain and fear in children receiving intravenous (IV) injections, cannulation and venepuncture. Although use of VR distraction is becoming more widespread in some disciplines (such as paediatric oncology/A&E), it is yet to be widely adopted in paediatric rheumatology.

**Case report - Case description:**

We report the case of a 9-year-old boy (ME) who in early 2019 developed psoriatic juvenile idiopathic arthritis (JIA) and an associated severe, widespread plaque psoriasis causing him substantial discomfort and distress. He has been treated with subcutaneous adalimumab biosimilar 40 mg fortnightly and oral methotrexate 20 mg weekly, alongside Keppra 1000 mg twice daily for epilepsy. Although he is just tolerating the subcutaneous adalimumab, he is requiring intermittent steroid joint injections under general anaesthetic for recurrent flares of his polyarticular arthritis and regular monitoring phlebotomy. He has been developing progressive needle phobia over the past 8 months, with superimposed anxiety about general anaesthetics with particular fear of the anaesthetic provoking an epileptic seizure.

Despite psychological intervention to teach coping strategies and standard play therapy distraction techniques, both phlebotomy and cannulation for general anaesthetics have become more distressing and challenging for him. During two recent day case admissions for joint injections, despite premedication with sedatives, distraction techniques and psychology interventions, he became so distressed that attempts to anaesthetise him had to be abandoned. This has resulted in prolonged periods of continuing joint inflammation and pain.

Discussion with local paediatric oncology colleagues resulted in a suggestion of using recently acquired VR headsets as an effective distraction technique. He has had a previous positive experience of using VR headsets in a play environment. He is receiving VR training intervention from the oncology team and is now fully engaged with the idea of using VR to distract him from negatively focussing on his next planned general anaesthetic intervention.

**Case report - Discussion:**

Unmanaged needle-related procedural pain in children is associated with increasing pain and stress in subsequent procedures and fear/avoidance of medical care which can affect compliance in chronic disease management. The psychological distress for children with needle phobia can add to existing psychological impact from JIA and increase family stress of caring for an ill child. The cost of poor management of needle-phobic children can be considerable, with cancelled or increased time for procedural interventions and restricted therapeutic interventions. Needle phobias in childhood may persist into adulthood.

Employing distraction techniques when children undergo medical procedures can help them reduce anxiety. VR is a method of captivating an individual’s interest in a virtual environment and can help divert attention from potentially distressing situations in the real world. Recent studies have reported VR as an effective distraction technique in children. Chan (2019) reports that VR significantly decreased the pain and post procedural anxiety experienced by children aged 4—11 years old during venepuncture/IV cannulation compared to controls. The majority of children in the VR group expressed interest in utilising VR again in the future, to improve their experience of IV injections. Chen (2020) subsequently reported a randomised controlled trial which significantly reduced pain and fear experienced from IV injections by the children in the VR group.

There are cost implications to both buying VR headsets/software and training play specialists/specialist nurses to use the equipment effectively. Furthermore, the acceptability of using VR with regards to the child’s age, engagement with age-appropriate software packages and whether a passive VR or a more interactive ‘gaming’ experience is preferred should be considered. The use of VR is not a panacea, but is potentially effective as an additional new tool in clinicians’ efforts to reduce the impact of needle phobia.

**Case report - Key learning points:**

Addressing needle phobia in children can require novel strategies.

VR has shown success as a distraction technique to alleviate pain and fear in children undergoing needle related procedures.

The implementation of VR in paediatric rheumatology has cost and staff training implications.

The use of age-appropriate software and an individual child’s preference for passive/active interaction with VR should be considered.

